# Supplementary material for: PCR Array Profiling of miRNA Expression Involved in the Differentiation of Amniotic Fluid Stem Cells toward Endothelial and Smooth Muscle Progenitor Cells
Source: Int J Mol Sci. 2023 Dec 25;25(1):302. doi: 10.3390/ijms25010302 (PMC10779355; doi:10.3390/ijms25010302)
Supplement: Supplementary file 1 [file ijms-25-00302-s001.zip › ijms-2735368-supplementary.pdf]

Table S1. General electrical characteristics of SPC from the n=24 whole-cell patch-clamp experiments and applied pharmacological compounds

Exp. no.	Cm (pF)	Rm (GOhms)	Ra (MOhms)	Tau (µs)	Hold (pA)	Pipette sol	Comp1.	Comp2.	Comp3.	Comp4.	Comp5.
19812	18.56668	5.082514	9.910785	183.636	0.087193	efficient					
a9812	53.65738	0.238585	9.236898	477.148	-1.75839	efficient					
b9812	102.5927	0	19.3656	1566.091	-1.75355	efficient	isoprenaline 1uM (sw.4-6)				
19813	19.98872	3.439259	18.31221	363.8603	7.706899	efficient					
a9813	22.22567	0.02929	12.1516	268.8575	127.7693	efficient					
b9813	89.67826	3.774866	37.55514	3333.431	-1.79472	efficient	phrixotoxin-1 280 nM (b9813006-007)	isoprenaline 1 uM (b9813008)	b9813010 sw.1-3 isopren.1uM; sw.4-10CTRL; sw.11-18 phrixotoxin1-280nM; sw.19-22 CTRL		
19814	13.26327	9.414673	8.058297	106.7609	1.019675	efficient+B-escin30uM	DIDS 100 uM	isoprenaline 1 uM			
a9814	10.03194	0.738725	50.90118	477.5226	-9.08988	efficient+B-escin30uM					
b9814	47.59146	5.126311	6.271327	298.0778	-3.70571	efficient+B-escin30uM	DIDS 100 uM (b9814006)	isoprenaline 1 uM (b9814006)			
19815	46.72194	0.337481	16.20167	721.9554	-171.623	efficient+B-escin30uM					
a9815	12.20971	5.351932	5.313883	64.80184	-5.46168	efficient+B-escin30uM					
b9815	14.25296	1.038694	8.21407	116.1505	-40.4552	efficient+B-escin30uM	iberiotoxin 100 nM (b9815001 sw.4-12)	isoprenaline 1 uM (b9815003-005)			
19821	28.66045	5.8211	5.259459	150.5858	1.295786	efficient	nifedipine 1uM (19821004-005)	mibefradil 5 uM (19821006-007;008)			
a9821	23.03082	4.831777	15.7046	360.4406	-1.82137	efficient	nifedipine 1uM	mibefradil 5 uM			
b9821	29.08172	0.119746	9.406697	253.6306	-72.4284	efficient	nifedipine 1uM (b9821004-006)	mibefradil 5 uM (b9821007-009)			
19822	19.73436	2.712283	22.58532	441.4361	-14.5855	efficient	NaCN 5 mM (19822006 sw.7-12)	glibenclamide 100 uM (19822006 sw.13-18)	DIDS 100 uM (19822006 sw.19-20)		
a9822	37.59555	0.342509	9.341733	341.8629	-18.2403	efficient	NaCN 5 mM	glibenclamide 100 uM	DIDS 100 uM		
b9822	33.23796	4.623733	4.336124	143.9814	-0.51347	efficient	NaCN 5 mM	glibenclamide 100 uM	DIDS 100 uM		
19904 *	27.61662	8.205615	13.25584	365.4303	1.261878	efficient	NaCN 5 mM				
a9904 *	31.38796	3.23914	20.57461	641.6375	-6.94638	efficient				Nifedipine 1uM (a9904008)	Mibefradil 10uM (a9904009)
b9904 *	27.96927	0.570465	7.877897	217.3263	-2.26702	efficient	NaCN 5 mM (b9904008-009)				
19909 *	38.3887	2.286035	16.83559	641.3249	-6.49588	efficient	NaCN 5 mM				
a9909 *	47.3762	5.394057	4.495904	212.7809	-0.07266	efficient	NaCN 5 mM				
b9909 *	43.96655	3.218766	8.215519	360.2402	-1.26672	efficient	NaCN 5 mM				
**Average**	**34.95112**	**3.164065**	**14.14091**	**504.5404**	**-9.214108517**						
**SD**	**22.58335**	**2.685901**	**10.86953**	**676.1914**	**47.30554816**						

* - SPC cultures pretreated with nifedipine 1 mM applied directly to the culture medium for 24-48 h

Table S2. Analysis of principal ionic current components recorded in SPC within the n = 24 whole-cell patch-clamp experiments

Exp. no.	I A current (pA) at +60 mV	tau at +60 mV (ms)	outward rectifier (pA) at +60 mV (average of last 100 ms)	BK-like current fluctuations at +60 mV		Na current		outward current at +80 mV (2.030-+0.07s)		inward rectifier	reversal potential (asc. ramp)
				peak ampl (pA)	SD (last 250 ms)	peak ampl. (pA)	voltage step of peak	mean (pA)	SD (pA)	pA at -120 mV (base at -80 mV)	mV
19812								74.0487	7.64698	-16.8	-46.5
a9812	596.3	4.63876	240.166	36.3	8.79673			222.172272	8.810999	-18.6	-66.9
b9812	2469	6.88189	555.942	127.3	34.4758			854.371826	19.862598	-42.1	-94.9
19813	50.32	3.02378	15.88	43.8	7.45868	-32.6	0	39.393467	13.509698	-4.3	-68.3
a9813											
b9813	210.9	7.43316	77.791	35.1	8.60377						
19814	391.5	13.9295	17.6061	29.3	5.34562			42.64294434	13.58994007	-3.4	-56.88
a9814											
b9814	-	-	88.4995	113.2	31.539			339.7509155	57.75685883	-5.5	-53.28
19815	-	-	454.533	49.4	14.373						
a9815								145.3754272	25.1792469	-	-36.67
b9815	170.6	14.3085	6.18702	25.3	3.60635			289.8139038	48.3679924	-17.1	-26.98
19821	-	-	121.004	138.9	29.8054			323.9849243	59.25339127	-41.5	-37.5
a9821	-	-	45.0157	65.9	13.4368			86.2383194	19.70644379	1.2	-72.2
b9821	510.6	26.4091	221.445	87.3	26.7935			451.7289429	36.21746063	-90	-42.24
19822	-	-	78.9054	62	13.7973			73.98690033	1.044837236	-16.2	-29.05
a9822	524.6	34.1908	198.322	144	34.1872			74.03349304	1.016172171	-42.18	-34.15
b9822	93.1	118.715	55.9557	97.9	21.5797			123.6133881	36.69259262	-6.5	-35.13
19904	-	-	27.396	37.3	10.9171			74.08636475	1.008280873	-3.6	-49.44
a9904	-	-	51.1314	86.1	20.1113			85.43177795	24.567379	-3.7	-51.71
b9904	119.6	73.4293	97.8734	104.1	26.4308			288.1426086	56.10247421	-7.9	-94.8
19909	-	-	103.275	82.9	26.6338			238.3852692	40.49472809	-11.6	-41.63
a9909	-	-	158.183	101.3	31.0576			312.6074524	64.26789856	-7.3	-84.38
b9909	-	-	141.104	118.7	33.886			251.4390564	50.62667465	-4.6	-89.75
**average**	**513.652**	**30.295979**	**137.810761**	**79.305**	**20.1417725**			**219.5623977**	**29.28613231**	**-17.9831579**	**-55.6195**
**SD**	**715.031**	**37.572358**	**143.441984**	**38.339048**	**10.7132904**			**192.0598567**	**21.39648512**	**22.12896168**	**22.05950982**

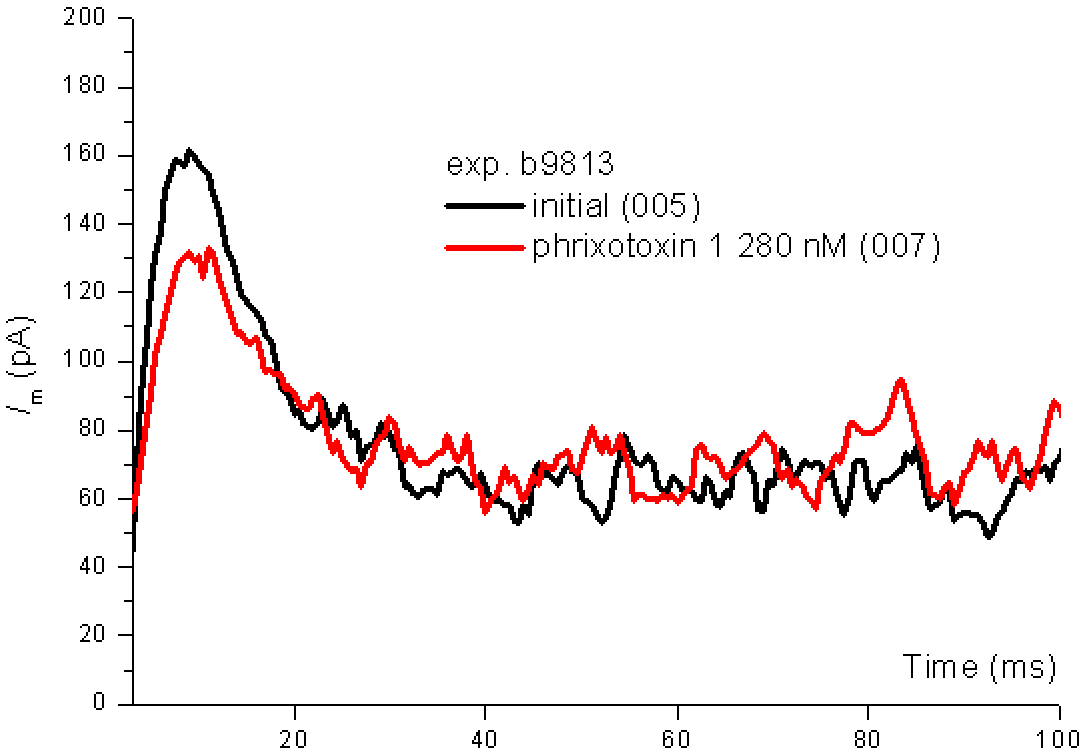

Figure S1. Inhibitory effect of phrixotoxin 1 (280 nM) on the I_{to} fast component, highlighted in the general IK voltage-clamp protocol during the depolarization step at +50 mV.

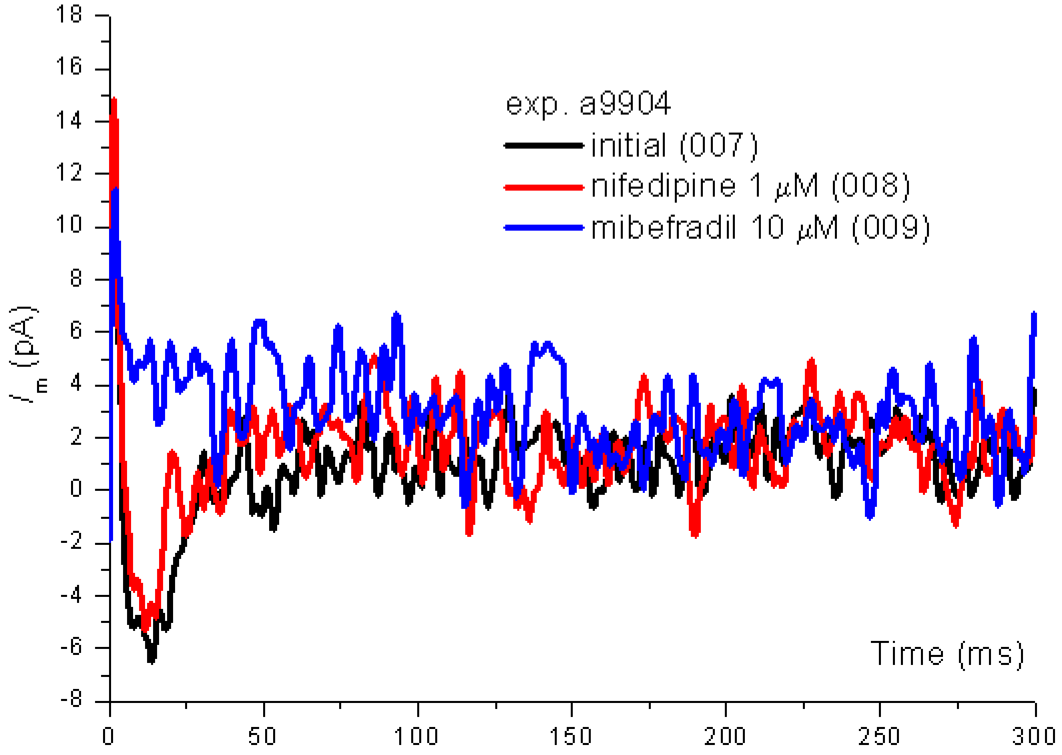

Figure S2. Lack of effect of 1 µM nifedipine and inhibitory effect of 10 µM mibefradil on the T-type Ca2+ current component (I_{CaT}) measured in the depolarizing step at -40 mV with the general IK protocol

Table S3. TaqMan assays used for characterization of progenitor cells.

Gene	TaqMan assay code	Producer
CD31 (PECAM-1)	Hs01065279_m1	Thermo Fischer Scientific, Massachusetts, USA
eNOS	Hs01574665_m1	Thermo Fischer Scientific, Massachusetts, USA
CD144 (VE-cadherin)	Hs00901465_m1	Thermo Fischer Scientific, Massachusetts, USA
CD54 (ICAM-1)	Hs00164932_m1	Thermo Fischer Scientific, Massachusetts, USA
vWF (von Wilebrand Factor)	Hs01109446_m1	Thermo Fischer Scientific, Massachusetts, USA
Smoothelin	Hs01022255_g1	Thermo Fischer Scientific, Massachusetts, USA
Calponin 1	Hs00959434_m1	Thermo Fischer Scientific, Massachusetts, USA
SMA (α- smooth muscle actin)	Hs00426835_g1	Thermo Fischer Scientific, Massachusetts, USA
Myh11 (myosin heavy chain)	Hs00975796_m1	Thermo Fischer Scientific, Massachusetts, USA
tropomyosin 1	Hs04398572_m1	Thermo Fischer Scientific, Massachusetts, USA
Caldesmon-1	Hs00921987_m1	Thermo Fischer Scientific, Massachusetts, USA
CACNA1G (cav3.1, R-type)	Hs00367969_m1	Thermo Fischer Scientific, Massachusetts, USA
